# Bortezomib attenuates HIF-1- but not HIF-2-mediated transcriptional activation

**DOI:** 10.3892/ol.2015.3545

**Published:** 2015-07-29

**Authors:** NORAINI ABD-AZIZ, ERIC J. STANBRIDGE, NORAZIZAH SHAFEE

**Affiliations:** 1 Department of Microbiology, Faculty of Biotechnology and Biomolecular Sciences, Universiti Putra Malaysia (UPM), Serdang 43400, Malaysia; 2 Department of Microbiology and Molecular Genetics, School of Medicine, University of California, Irvine, CA 92697, USA

**Keywords:** bortezomib, hypoxia-inducible factor, HIF-1, HIF-2, transcriptional activity

## Abstract

Bortezomib is the first proteasomal inhibitor (PI) to be used therapeutically for treating relapse cases of multiple myeloma and mantle cell lymphoma. A proposed mechanism for its action is that it prevents the proteasomal degradation of proapoptotic proteins, leading to enhanced apoptosis. Although the α subunit of hypoxia-inducible factor (HIF)-1 is not degraded with bortezomib treatment, the heterodimeric HIF-1 fails to transactivate target genes. HIF-1 and HIF-2 are related hypoxia-inducible transcription factors that are important for the survival of hypoxic tumor cells. The majority of reports have focused on the effects of bortezomib on the transcriptional activities of HIF-1, but not HIF-2. The present study investigated the effects of bortezomib on HIF-2 activity in cancer cells with different levels of HIF-1α and HIF-2α subunits. HIF-α subunit levels were detected using specific antibodies, while HIF transcriptional activities were evaluated using immunodetection, reverse transcription-polymerase chain reaction and luciferase reporter assay. Bortezomib treatment was found to suppress the transcription and expression of *CA9*, a HIF-1-specific target gene; however, it had minimal effects on *EPO* and *GLUT-1*, which are target genes of both HIF-1 and HIF-2. These data suggest that bortezomib attenuates the transcriptional activity only of HIF-1, and not HIF-2. This novel finding on the lack of an inhibitory effect of bortezomib on HIF-2 transcriptional activity has implications for the improvement of design and treatment modalities of bortezomib and other PI drugs.

## Introduction

Bortezomib (also identified as PS-341 or Velcade®) is the first proteasomal inhibitor (PI) to be utilized for cancer therapy; it is used to treat multiple myeloma and mantle cell lymphoma (1). Bortezomib has been demonstrated to inhibit tumor neoangiogenesis, a requirement for cancer progression and metastasis (2). This inhibition is accomplished through the upregulation of proapoptotic proteins and the suppression of pathways responsible for antiapoptotic gene expression (3). Bortezomib has also been demonstrated to obstruct hypoxia adaptation in tumors by repressing the activity of hypoxia-inducible factor (HIF)-1, a transcription factor (4,5). HIF-1 is a heterodimer, composed of an oxygen-regulated α and a constitutively expressed β subunit.

HIF-1 is one of three related heterodimeric hypoxia-inducible factors (HIF-1, −2 and −3), which possess a common β subunit and differing α subunits. The α subunit protein in each case is rapidly degraded under normoxic conditions, but is stable under hypoxic conditions. HIF-1 and HIF-2 have been revealed to play important roles in the survival of hypoxic cells in solid tumors (6). HIF-1α is structurally similar to HIF-2α; these two subunits share 48% amino acid sequence identity and are regulated in a similar manner (7). However, despite these similarities, the heterodimeric HIF-1 and HIF-2 proteins exhibit distinct functional roles in cancer. They also transactivate a number of common as well as distinct downstream target genes (8). For example, HIF-1, but not HIF-2, specifically regulates the transcription of carbonic anhydrase 9 (*CA9*) (9) and phosphoglycerate kinase (*PGK*) (8,10). By contrast, other hypoxia-inducible genes, including glucose transporter-1 (*GLUT-1*) and erythropoietin (*EPO*), are targets of HIF-1α and HIF-2α (8,10–12). In addition to differing in terms of target genes, the expression levels of the α subunits of HIF-1 and HIF-2 also vary in cells and tissues. In neuroblastoma cells expressing HIF-1α and HIF-2α in normoxic levels of oxygen, the HIF-1α protein has been demonstrated to be expressed at a much lower level compared with HIF-2α (13).

To date, mechanistic studies of the effects of bortezomib on HIF have predominantly focused only on the inhibition of proteasomal degradation of HIF-1α (5,14). Previously, our group and others demonstrated that bortezomib treatment led to an accumulation of HIF-1α; however, the corresponding increased level of heterodimeric HIF-1 was inactive (4,15). To the best of our knowledge, no studies have investigated the effects of bortezomib on HIF-2 transcriptional activity. Therefore, in the present study, the effects of bortezomib treatment on the stabilization of HIF-2α and corresponding HIF-2 activity were examined using cancer cell lines known to express both HIF-1α and HIF-2α, and a cancer cell line expressing only HIF-2α.

## Materials and methods

### 

#### Human cell lines and culture

Osteosarcoma (Saos-2), breast carcinoma (MCF-7) and renal clear cell carcinoma (786-O) cell lines were obtained from the American Type Culture Collection (Manassas, VA, USA) and maintained in Dulbecco's modified Eagle's medium supplemented with 10% fetal bovine serum (GE Healthcare, Pasching, Austria). Cells were grown in normoxic conditions (21% O_2_) in a humidified Forma 311 CO_2_ incubator (Thermo Forma, Marietta, OH, USA), or hypoxic conditions (0.5% O_2_) in a Galaxy 48R incubator (New Brunswick™, Eppendorf, Hamburg, Germany). The PI bortezomib (Millennium Pharmaceuticals, Inc., Cambridge, MA, USA) was dissolved in dimethyl sulfoxide. For bortezomib treatment, cells were initially seeded at 6.6×10^4^ cells/cm^2^ for 24 h. Cells were pre-treated with bortezomib for 30 min, and then exposed to normoxia or hypoxia for 24 h in the presence of the drug (4). IC_20_ concentrations of bortezomib (0.5, 0.2 and 0.17 µM for Saos-2, MCF-7 and 786-O, respectively) were used. Samples were harvested on ice using radioimmunoprecipitation assay buffer (Thermo Fisher Scientific, Inc., Rockford, IL, USA) containing EDTA-free protease inhibitor cocktail (Roche, Mannheim, Germany). Samples were probed using antibodies against HIF-1α (monoclonal rabbit anti-human; cat. no. GTX61608; 1,1,000), HIF-2α (monoclonal rabbit anti-human; cat. no. GTX103707: 1:1,000), GLUT-1 (polyclonal rabbit anti-human; cat. no. GTX100684; 1,1,000), carbonic anhydrase IX (CAIX; monoclonal mouse anti-human; cat. no. GTX70020; 1,1,000) (all from Genetex, Inc., Irvine, CA, USA), EPO (polyclonal rabbit anti-human; cat. no. sc-7956; 1,1,000; Santa Cruz Biotechnology, Inc., Dallas, TX, USA) and β-actin (monoclonal mouse anti-human; cat. no. A5316; 1:5,000; Sigma-Aldrich, St. Louis, MO, USA) for 1 h at room temperature. The samples were washed three times with Tris-buffered saline containing 0.1% Tween 20 (Amresco LLC, Solon, OH, USA), then probed with horseradish peroxidase-conjugated monoclonal horse anti-mouse (cat. no. 7076S; 1:5,000) or polyclonal goat anti-rabbit (cat. no. 7074S; 1:5,000) IgG secondary antibodies (Cell Signaling Technology, Inc., Danvers, MA, USA) for 1 h at room temperature. Protein bands were detected using the SuperSignal West Dura Extended Duration Substrate kit (Pierce Biotechnology, Inc., Rockford, IL, USA)and quantitated using ImageJ software (version 1.48; National Institutes of Health, Bethesda, MD, USA) as previously described (16).

#### Reverse transcription-polymerase chain reaction (RT-PCR)

RT-PCR was performed on 100 ng of RNA using the Access RT-PCR system (Promega Corporation, Madison, WI, USA). Specific primers for *HIF*-1α (4,17), *HIF-2*α (18), *CA9* (4), *GLUT-1*, *EPO* (19) and β-*actin* (4) were used. The reaction system (Access RT-PCR system; Promega Corporation) contained 1X AMV/Tfl Reaction buffer, 10 mM dNTP mix, Tfl DNA polymerase (0.1 U), AMV RT (0.1 U), 25 mM MgSO_4_, 10 mM forward and reverse primers. PCR was performed under the following conditions: 1 cycle of reverse transcription at 45°C for 45 min, 1 cycle of predenaturation at 94°C for 2 min, followed by 30 cycles (with the exception of β-actin, 25 cycles) at 95°C for 40 sec, 56°C for 40 sec followed by 72°C for 1 min with a final extension step at 72°C for 4 min. RT-PCR products were then analyzed on 1.5% agarose gel and quantitated using ImageJ 1.48 software.

#### Luciferase reporter assay

Transfection with a firefly luciferase reporter construct driven by the hypoxia response elements (HREs) of *CA9*, *PGK* and *EPO* was performed using the pLuc-MCS vector (Agilent Technologies, Inc., Santa Clara, CA, USA) and Lipofectamine 2000 (Invitrogen Life Technologies, Carlsbad, CA, USA) as previously described (20). The HRE sequences are 5′-GGCTGTACGTGCATTGGAAACGAGAGCTG for *CA9*, 5′-TTTGTCACGTCCTGCACGACGCG for *PGK* and 5′-GGCCCTACGTGCTGTCTCACACAGCCTGT for *EPO*. A non-hypoxia-responsive plasmid, pRL-CMV (Promega Corporation), expressing *Renilla* luciferase was used as the internal control as described previously (20). Luciferase activities were determined using a Dual-Luciferase® Reporter Assay System (Promega Corporation) in a Sirius luminometer (Titertek-Berthold, Pforzheim, Germany), according to the manufacturer's instructions. Data are presented as the average ratio of firefly to *Renilla* luciferase activities [± standard deviations [SD)] from at least three independent experiments.

#### Statistical analysis

Experimental data were analyzed using the Student's *t*-test (GraphPad Prism 5; GraphPad Software, Inc., La Jolla, CA, USA) and expressed as the mean ± standard error of the mean (SEM). P<0.05 was considered to indicate a statistically significant difference.

## Results and Discussion

### 

#### Bortezomib attenuates HIF-1 but not HIF-2 transcriptional activity

**Figure 1.**
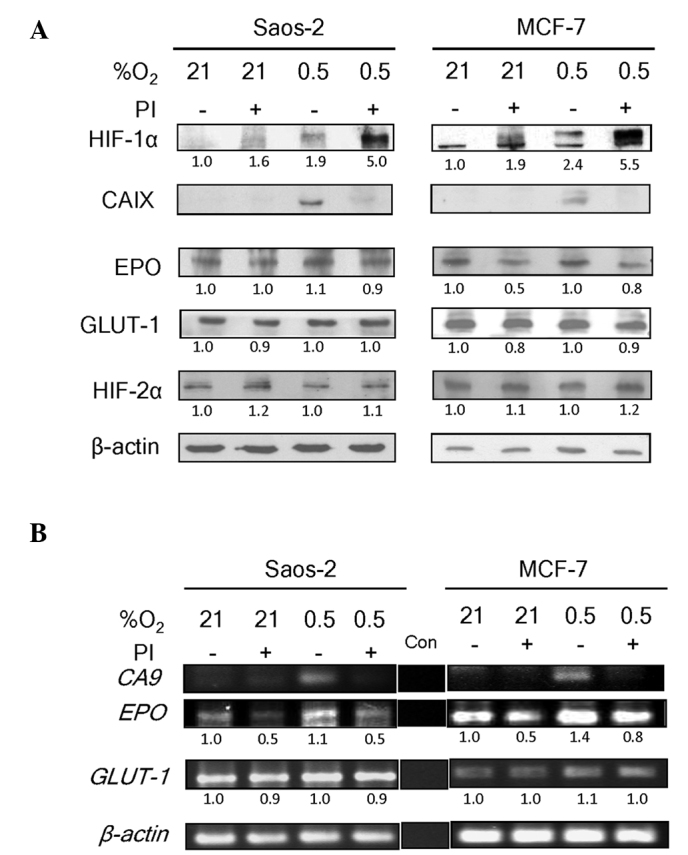
Bortezomib attenuates HIF-1 but not HIF-2 transcriptional activity. Saos-2 and MCF-7 cells were cultured under normoxia (21% O_2_) or hypoxia (0.5% O_2_) in the presence or absence of bortezomib. The levels of HIF-1α, HIF-2α and their (A) target gene expression and (B) their transcript levels were examined. PI, proteasomal inhibitor (bortezomib); HIF, hypoxia-inducible factor; CAIX, carbonic anhydrase IX; *CA9*, carbonic anhydrase 9; EPO, erythropoietin; GLUT-1, glucose transporter 1; Con, control (template replaced with water).

HIF-1α and HIF-2α subunits are closely related (21), however their hypoxic regulation, pattern of expression and specific target genes vary to a certain degree (8). Previously, our group demonstrated that bortezomib attenuated the transcriptional activity of HIF-1 in a number of cancer cell lines (4). Since the accumulated inactive HIF-1 still formed a complex with the coactivator p300, the involvement of a corepressor in its attenuated activity was proposed. p300 is an important component of the transcriptional machinery which is involved in the regulation of chromatin organization and transcription initiation (4). However, the exact mechanism involving the potential corepressor(s) remains a topic of investigation. In the present study, the attenuated effect of bortezomib on HIF-1 activity was reproduced in Saos-2 and MCF-7 cell lines. These cell lines express both HIF-1α and HIF-2α proteins (4,22). Bortezomib treatment caused an accumulation of HIF-1α protein under normoxia (21% O_2_), as well as further accumulation under hypoxia (0.5% O_2_) in the two cell lines (Fig. 1A). In normoxic conditions, bortezomib-mediated HIF-1α stabilization failed to cause upregulation of the CAIX protein, of which the encoding gene, *CA9*, is a HIF-1 specific target (9). As expected, the hypoxia-induced accumulation of HIF-1α in the absence of bortezomib was associated with an increase in CAIX expression. This expression was absent in the presence of bortezomib. These observations concur with those of our previous study, which showed stabilization of inactive HIF-1 with bortezomib treatment in Saos-2 and MCF-7 cell lines (4).

Notably, the levels of two other HIF-regulated proteins, EPO (11,12) and GLUT-1 (8,10) were only minimally reduced by bortezomib treatment (Fig. 1A). *EPO* and *GLUT-1* are regulated by HIF-1 as well as HIF-2 (8,10–12). As their expression patterns in response to bortezomib treatment differed from that of *CA9*, a HIF-1-specific target gene, we hypothesized that their continued expression in the presence of bortezomib was due to the lack of an inhibitory effect of the drug on HIF-2 activity. To test this, the level of HIF-2α in the samples was examined. HIF-2α was found to be expressed constitutively at high levels in Saos-2 and MCF-7 cells under the normoxic and hypoxic conditions used in the present study (Fig. 1A). This was consistent with the patterns of HIF-2α protein levels under normoxic conditions reported in another study (13). HIF-2α is less efficiently degraded via the prolyl-4-hydroxylase-mediated proteasomal degradation pathway compared with HIF-1α under physiological oxygen conditions (13). In the present study, the basal levels of HIF-2α in Saos-2 and MCF-7 cells were not increased by hypoxia, indicating that the constitutive levels approached saturation. In accordance with this, bortezomib treatment only marginally increased the level of HIF-2α protein under normoxic or hypoxic conditions.

#### Bortezomib does not inhibit HIF-2 transcriptional activity in 786-O cells

The effect of bortezomib on the transcriptional activities of HIF-1 was observed (4). To confirm that bortezomib interfered only with transcriptional activities of HIF-1 and not HIF-2, RT-PCR was performed using *CA9*-, *EPO*- and *GLUT-1*-specific primers. A band representing the *CA9* transcript, which is exclusively under HIF-1 regulation (9), was visible only in hypoxic conditions in the absence of bortezomib (Fig. 1B). No *CA9* band was observed in the normoxic conditions (without bortezomib), which was in accordance with the absence of HIF-1α (Fig. 1A). In all bortezomib-treated samples, despite the accumulation of HIF-1α (Fig. 1A), the *CA9* band was almost absent. The levels of *EPO* and *GLUT-1* transcripts, however, were clearly visible even under normoxia (Fig. 1B). As these genes are under the regulation of HIF-1 and HIF-2 (8,10–12), the result is consistent with a lack of effect of bortezomib on the functional status of constitutively expressed HIF-2α. The modest decrease in EPO band intensity is likely to reflect the inhibition of HIF-1 by bortezomib, as EPO is regulated by both HIF-1 and HIF-2. These varying effects of bortezomib concur with the concept that HIF-1 and HIF-2 have non-redundant roles in the regulation of their target genes (23). Therefore, the suppression of HIF-1 activity may not directly affect HIF-2 activity. Other cell lines are currently being investigated by our group to address the possibility of cell-type specific aspects of the findings.

**Figure 2.**
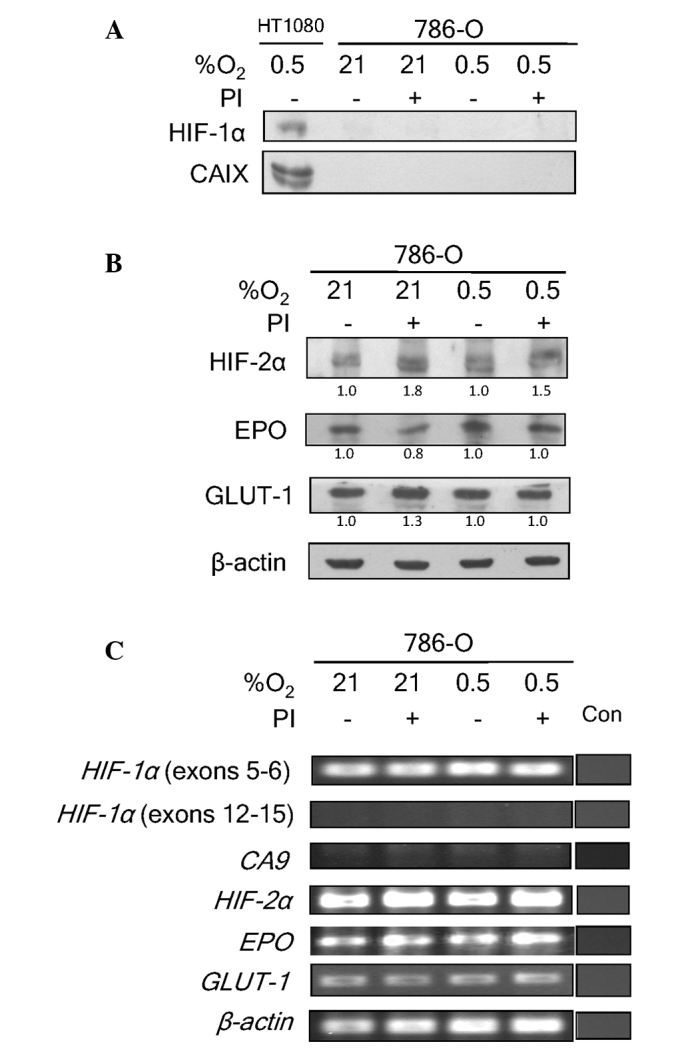
HIF-2 transcriptional activity in 786-O cells is not inhibited by bortezomib. 786-O cells were cultured in either 21% O_2_ or 0.5% O_2_ in the presence or absence of bortezomib. (A) HIF-1α and CAIX protein expression was not detected in the cells. No marked changes in HIF-2 transcriptional activity were noted, based on (B) protein and (C) transcript levels. PI, proteasomal inhibitor (bortezomib); HIF, hypoxia-inducible factor; *CA9*, carbonic anhydrase 9; EPO, erythropoietin; GLUT-1, glucose transporter 1; Con, control (template replaced with water).

To confirm that bortezomib did not attenuate the activity of HIF-2, the HIF-1α-deficient 786-O cell line was also examined (24). This cell line is devoid of the Von Hippel-Lindau (VHL) tumor suppressor (8), and therefore has a constitutive stabilization of HIF-2α. VHL forms a complex with elongin-B, elongin-C and cullin-2 to function as an E3 ubiquitin ligase for ubiquitination and degradation of hydroxylated HIF-α proteins (4). Since these cells express HIF-2α and not HIF-1α, they allow the investigation of the effects of bortezomib on HIF-2 exclusively. Predictably, no HIF-1α protein expression was detected in 786-O cells (Fig. 2A). The absence of HIF-1α in 786-O was associated with a lack of CAIX expression. The addition of bortezomib caused a marginal increase in HIF-2α expression under normoxic and hypoxic conditions (Fig. 2B). This increase, however, did not significantly influence the expression level of EPO or GLUT-1 proteins, which are also HIF-2 target genes. These data further strengthen the hypothesis that bortezomib does not interfere with HIF-2 transcriptional activity, as it does with HIF-1.

At the genetic level, the absence of exons 12–15 for *HIF-1a* was confirmed in 786-O cells (Fig. 2C). The absence of these exons has been previously reported (17). Exons 5–6, however, were still present. The lack of functioning HIF-1α resulted in the absence of transcriptional activation of *CA9* by HIF-1 in the cells (Fig. 2C). *HIF-2*α transcript levels, by contrast, were not significantly affected by hypoxia or bortezomib treatment. The transcript levels of *EPO* and *GLUT-1* also remained unaltered. The lack of functional HIF-1α in 786-O implied that *EPO* and *GLUT-1* expression was being regulated solely by HIF-2 in this cell line. These data clearly demonstrate a lack of influence of bortezomib on HIF-2 transcriptional activity. They further confirm the assertion of the differential effects of bortezomib on HIF-1 and HIF-2 transcriptional activities.

#### Bortezomib inhibits the activation of exogenously-introduced promoters of HIF-1, but not HIF-2 target genes

**Figure 3.**
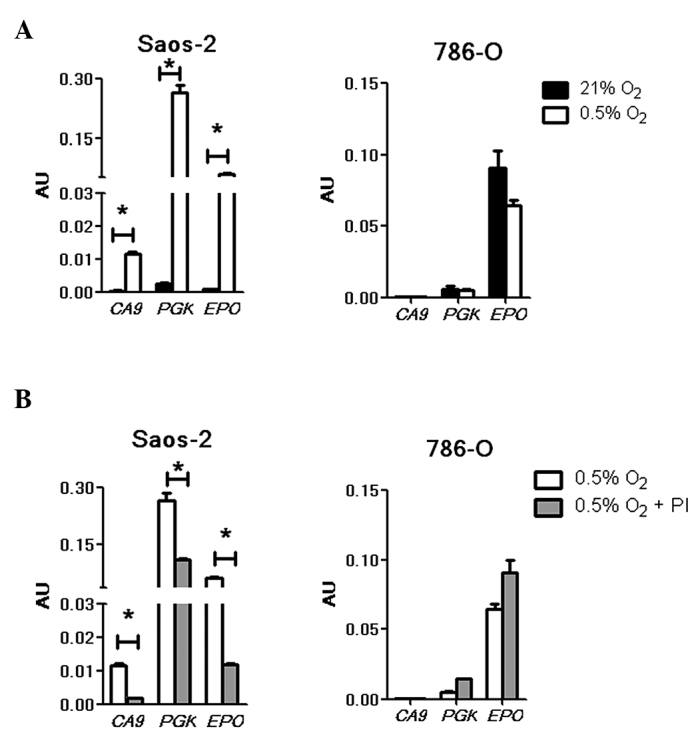
Activation of exogenously-introduced promoters of HIF-1, but not HIF-2 target genes, is inhibited by bortezomib. (A) Saos-2 and 786-O cells transfected with specific luciferase reporter constructs exhibited varying levels of hypoxia-inducible signals. (B) These signals were suppressed by bortezomib in Saos-2 cells but not in 786-O cells. *P<0.05. HIF, hypoxia-inducible factor; *CA9*, carbonic anhydrase 9; *PGK*, phosphoglycerate kinase; *EPO*, erythropoietin; PI, proteasomal inhibitor (bortezomib); AU, arbitary unit.

To investigate the effects of bortezomib treatment on exogenously introduced HREs of HIF-1 and HIF-2 target genes, a Dual-Luciferase® Reporter Assay was performed using selected plasmid constructs (4,20) carrying a *CA9* (regulated by HIF-1) or an *EPO* (regulated by HIF-1 and HIF-2) HRE. As the available *CA9* reporter construct produced low luciferase signals (20), a HRE construct of another HIF-1-specific target gene, *PGK*, was also included (8,10). All constructs were responsive to hypoxic stimuli in Saos-2 cells (Fig. 3A, left panel). The hypoxia-induced luciferase signal driven by the *CA9* HRE was low compared with that of *PGK*, as previously documented (20). In contrast to Saos-2, no activation of the *CA9* and *PGK* HRE constructs was observed in the hypoxic 786-O cells (Fig. 3A, right panel), indicating the absence of functional HIF-1 in the cells. Under normoxia and hypoxia, no signal was detected for *CA9; however*, a low level *PGK* signal was observed. The presence of PGK expression in 786-O cells has been previously reported (8,10) and, in accordance with the current findings, a basal level of expression that was not enhanced by hypoxia was documented. Unlike the *CA9* and *PGK* reporter constructs, *EPO* produced high luciferase signals under both normoxic and hypoxic conditions. Although a minimal reduction was noted under hypoxia, this was not statistically different from the normoxic results (P>0.05). These data were consistent with the previously reported constitutive expression of HIF-2α, and thereby constitutively active HIF-2, in this cell line (8).

To examine the effects of bortezomib on these HRE-driven luciferase signals, Saos-2 and 785-O cells were treated with the drug. In agreement with previous studies (4,20), the hypoxia-induced signals for *CA9*, *PGK* and *EPO* were markedly suppressed in the presence of bortezomib in hypoxic Saos-2 cells (P<0.05; Fig. 3B, left panel). These results indicate that HIF-1 transactivation of the *CA9* and *PGK* (HIF-1 target genes), as well as *EPO* (a HIF-1 and HIF-2 target gene) promoters were likely suppressed by the drug. In agreement with a previous report (4), the repression of *CA9* and *PGK* was not absolute. In 786-O cells, there was no detectable signal from the *CA9* HRE construct under either treatment condition (Fig. 3B, right panel). Additionally, a non-hypoxia-inducible background reading for *PGK* (10) was observed and was not reduced by the drug treatment. *EPO* HRE-driven luciferase expression, which was high in hypoxic conditions, was also not significantly affected by the drug. This result is further indication of the concept that bortezomib attenuates only HIF-1 and not HIF-2 transcriptional activities. This distinction may have profound clinical implications for certain cancer types. For example, overexpression of HIF-2α has a protumorigenic effect when HIF-1 activity is lacking (25). Furthermore, recent evidence demonstrated that HIF-1 is involved in the inhibition of cell proliferation via a non-transcriptional mechanism, while HIF-2 enhances cell proliferation (26). In line with our findings, it is tempting to speculate that bortezomib also inhibits this specific HIF-1 action, which would account for the reduced efficacy of bortezomib in certain cancers with differing levels of HIF-1/HIF-2. Our group is currently investigating this possibility.

In conclusion, using the Saos-2, MCF-7 and 786-O cell lines as models of cells with differing levels of HIF-1α and HIF-2α, the present study demonstrated the inhibitory effect of bortezomib on HIF-1 but not HIF-2 transcriptional activities. Even though the molecular mechanisms that underlie such specificity are yet to be elucidated, information obtained in the current study will contribute towards a further understanding of the therapeutic efficacy of bortezomib, and potentially of other PI drugs, for cancer cells that express HIF-1α and/or HIF-2α; a case in point is renal clear cell carcinoma (17).
